# Comparative Characterization of Mitogenomes From Five Orders of Cestodes (Eucestoda: Tapeworms)

**DOI:** 10.3389/fgene.2021.788871

**Published:** 2021-12-22

**Authors:** Bruna Trevisan, Denis Jacob Machado, Daniel J. G. Lahr, Fernando P. L. Marques

**Affiliations:** ^1^ Department of Zoology, Institute of Biosciences, University of São Paulo, São Paulo, Brazil; ^2^ Department of Bioinformatics and Genomics, College of Computing and Informatics, University of North Carolina at Charlotte, Charlotte, NC, United States

**Keywords:** parasitology, high-throughput sequencing, mitogenomics, gene order, molecular markers, informative sites

## Abstract

The recognized potential of using mitogenomics in phylogenetics and the more accessible use of high-throughput sequencing (HTS) offer an opportunity to investigate groups of neglected organisms. Here, we leveraged HTS to execute the most comprehensive documentation of mitogenomes for cestodes based on the number of terminals sequenced. We adopted modern approaches to obtain the complete mitogenome sequences of 86 specimens representing five orders of cestodes (three reported for the first time: Phyllobothriidea, “Tetraphyllidea” and Trypanorhyncha). These complete mitogenomes represent an increase of 41% of the mitogenomes available for cestodes (61–147) and an addition of 33% in the representativeness of the cestode orders. The complete mitochondrial genomes are conserved, circular, encoded in the same strand, and transcribed in the same direction, following the pattern observed previously for tapeworms. Their length varies from 13,369 to 13,795 bp, containing 36 genes in total. Except for the Trypanorhyncha specimen, the gene order of the other four cestode orders sequenced here suggests that it could be a synapomorphy for the acetabulate group (with a reversion for taenids). Our results also suggest that no single gene can tell all the evolutionary history contained in the mitogenome. Therefore, cestodes phylogenies based on a single mitochondrial marker may fail to capture their evolutionary history. We predict that such phylogenies would be improved if conducted under a total evidence framework. The characterization of the new mitochondrial genomes is the first step to provide a valuable resource for future studies on the evolutionary relationships of these groups of parasites.

## Introduction

Cestodes, commonly known as tapeworms, are composed of more than 5,000 cosmopolitan species. They are primarily endoparasites in the digestive tract of vertebrates when adults (Stunkard, 1962; Caira and Reyda, 2005; Caira et al., 2017). This diverse group of Metazoa is organized into 19 orders (Caira et al., 2012; Caira and Littlewood, 2013; Caira et al., 2014b; Caira et al., 2017), nine of which parasitize the spiral intestines of elasmobranchs and possess a long evolutionary history with their hosts (Caira et al., 2014a).

The systematics of Cestoda has been restructured during the last decades, mainly due to new evidence provided by phylogenetic hypotheses based on molecular data (Mariaux, 1998; Olson et al., 1999; de Chambrier et al., 2004; Caira et al., 2005; Kuchta et al., 2007; Kuchta et al., 2008; Healy et al., 2009; Caira et al., 2014a; Brabec et al., 2015; Trevisan et al., 2017; Caira et al., 2020). The current concept of the Rhinebothriidea Healy et al. (2009) is one of the examples of reformulation in cestode systematics. Support for the monophyly of the order is based on the phylogenetic analyses of nucleotide sequences (i.e., 18S and partial 28S) (Healy et al., 2009; Caira et al., 2014a; Trevisan et al., 2017), but its generic composition is still under debate (see Ruhnke et al., 2017).

Despite recent advances, cestode systematics have been restricted to few molecular markers and low taxonomic representation. The majority of studies published thus far are based on molecular data from partial sequences of two RNA nuclear markers (i.e., 18S and 28S; see Healy et al., 2009; Ruhnke et al., 2015; Trevisan et al., 2017; Waeschenbach and Littlewood, 2017; Golzarianpour et al., 2020). Few, however, have used only one mitochondrial marker (COI in Reyda and Marques, 2011), which limits the power to test phylogenetic hypotheses (León and Nadler, 2010). In addition, compared to the diversity of the group, most studies have low taxonomic representation (e.g., Healy et al., 2009; Reyda and Marques, 2011; Caira et al., 2014a; Ruhnke et al., 2015; Trevisan et al., 2017; Golzarianpour et al., 2020). These two components limit our understanding of the phylogenetic relationships among cestodes.

Since the beginning of the popularization of molecular data, the mitogenome is recognized as a rich source of information that can be used as a molecular marker in evolutionary studies (Avise et al., 1987; Le et al., 2000; Hebert et al., 2003; Ballard and Whitlock, 2004; Lefébure et al., 2006; Park et al., 2007; Zarowiecki et al., 2007; Jex et al., 2010; Avise, 2012; Cameron, 2014; Ladoukakis and Zouros, 2017; Li et al., 2017; Li JY. et al., 2019; Tan et al., 2019; Trevisan et al., 2019; Landeryou et al., 2020). The potential of using the mitogenome is related to its conserved genetic content with elevated mutation rate compared to nuclear DNA, which allows us to identify and assign specimens to known taxonomic groups (Crozier, 1990; Hebert et al., 2003; Lefébure et al., 2006; Ratnasingham and Hebert, 2007; Cameron, 2014; Ladoukakis and Zouros, 2017; Li et al., 2017). In addition to taxonomic identification, regions of the mitogenome—especially the Cytochrome Oxidase I (MT-CO1)—have been traditionally used for phylogenetic and phylogeographic inference in the past decades (Wilson et al., 1985; Avise et al., 1987; Moritz et al., 1987; Crozier, 1990; Palumbi and Wilson, 1990; Hillis et al., 1996; Frohlich et al., 1999; Le, et al., 2000; Hebert et al., 2003; Lefébure et al., 2006; Zarowiecki et al., 2007; Avise, 2012).

One of the reasons for the popularization of the MT-CO1 over the years is the availability of universal primers and its high mutation rate. Within Sanger sequencing protocols, developing primers for new molecular markers is time-consuming and involves high cost and risk without a guaranteed return. That led researchers to adopt mitochondrial primers from previous studies that addressed similar questions ignoring whether they represent the most suitable marker for particular research (Zarowiecki et al., 2007). Accessing the complete mitochondrial genome under Sanger sequencing protocols would be a costly and time-consuming alternative to deviate from the traditional markers used from this genome.

The limitations of primer design and access to whole organelle genomes have been addressed with the emergence of new technologies. The development of high throughput sequencing (HTS) enables the sequencing and analysis of molecular data from non-model organisms on an unprecedented scale. As such, HTS made the acquisition of complete genomes economically feasible in a reasonable time (Mardis, 2006; Schuster, 2008; Jex et al., 2010; Metzker, 2010; Zhang et al., 2011; Straub, et al., 2012; Hahn et al., 2013; Gan et al., 2014; Pons et al., 2014; Grandjean et al., 2017; Machado et al., 2016; Li et al., 2017; Tang et al., 2017; Li et al., 2018; Bondarenko et al., 2019; Li W. X. et al., 2019; Tan et al., 2019; Trevisan et al., 2019; Landeryou et al., 2020; Lin et al., 2020). Among the HTS methods available to date, “genome skimming” consists of sequencing total genomic DNA with low coverage, generating many copies of fractions of the complete genomic DNA from organelles, such as mitochondria and from nuclear ribosomal DNA (Straub et al., 2012; Grandjean et al., 2017; Trevisan et al., 2019). The main advantage of sequencing the total genomic DNA is that it does not require PCR amplification and prior knowledge of molecular markers, which minimizes errors and avoids problems in the primer design process. Thus, this approach appears as a potential solution to recover the complete mitogenome in non-model groups, solving the limitations of restricted molecular markers (Grandjean et al., 2017; Trevisan et al., 2019; Landeryou et al., 2020; Lin et al., 2020).

To date, there are 61 cestodes mitogenomes publicly available at NCBI (Supplementary Table S1) (Le et al., 2000; von Nickisch-Rosenegk et al., 2001; Nakao et al., 2002; Nakao et al., 2003; Jeon et al., 2005; Jeon et al., 2007; Nakao et al., 2007; Park et al., 2007; Jia et al., 2010; Liu et al., 2011; Yamasaki et al., 2012; Nakao et al., 2013; Terefe et al., 2014; Eom et al., 2015; Guo, 2015; Brabec et al., 2016; Guo, 2016; Cheng et al., 2016; Lavikainen et al., 2016; Wang et al., 2016; Zhao et al., 2016; Gao et al., 2017; Li. et al., 2017; Tang et al., 2017; Li et al., 2018; Xi et al., 2018; Li W. X. et al., 2019; Trevisan et al., 2019). In total, they represent six of the 19 orders currently recognized for Cestoda (Caira et al., 2017). Most mitogenomes (91.6%) were assembled using Sanger technology. The majority of them are from members of Cyclophyllidea (67.2%) due to its economic relevance. Mitogenomes from other cestode orders (including the Rhinebothriidea) are scarce (Littlewood et al., 2008; Maldonado et al., 2017). Therefore, additional effort is necessary to fill the lack of information for many taxonomic groups, which potentially can contribute to our understanding of the evolution of this group and may help elucidate some hitherto historically unsolved issues in the systematics of cestodes (e.g., the composition of “Tetraphyllidea” or *Rhinebothrium*; taxa consistently found to be non-monophyletic).

Despite recent efforts to sequence mitogenomes from cestodes, we are far from documenting its diversity adequately throughout this group of parasites. Here, we present 86 new mitogenomes of five orders of cestodes parasites of elasmobranchs, emphasizing the Rhinebothriidea. We used HTS data based on genome skimming from an unprecedented taxonomic sample. The method used allowed the acquisition of data for multiple specimens in a single sequencing run, successfully recovering their complete mitogenomes. The characterization of these new mitochondrial genomes is the first step to provide a helpful source for future studies on the evolutionary relationships of these groups of parasites.

## Materials and Methods

### Sampling and DNA Extraction

We examined a total of 45 specimens of stingrays representing 25 species. Details on hosts and sampling are in the supporting information (Supplementary Table S2). Stingrays were collected using available fishing gear for each locality (e.g., handheld spears, spear guns, or handheld lines, landlines, or long-lines) and following the guidelines of collecting permits issued by local authorities.

The parasite specimens were removed from the spiral intestines of their hosts, fixed in 96% ethanol, and stored at –20°C. We took tissue samples from the middle portion of the strobila of each specimen following the protocols commonly used for cestodes (Trevisan et al., 2017; Trevisan et al., 2019). Following the manufacturer’s instructions, we extracted the total genomic DNA from the tissue samples using Agencourt’s DNAdvance Nucleic Acid Isolation Kit (Beckman Coulter, Brea, CA, United States). Protocols to avoid contamination were taken as suggested by Trevisan et al. (2019). We deposited hologenophores (sensu Pleijel et al., 2008) at MZUSP (Museu de Zoologia da Universidade de São Paulo, Universidade de São Paulo, São Paulo, SP, Brazil) and LRP (Lawrence R. Penner Parasitology Collection, University of Connecticut, Storrs, Connecticut, United States). For more details on host collection and specimen preparation, see Trevisan et al. (2017) and Marques and Reyda (2015).

### Library Preparation and Mitogenome Sequencing

We chose Nextera XT DNA Library Preparation Kit (Illumina) to prepare indexed paired-end (PE) libraries, following Trevisan et al. (2019). This protocol is suitable for DNA extractions of non-model taxa with different ages of fixation and requires small amounts of input DNA. The new protocol and strategies that the authors proposed were also followed here as 0.2 ng/ul as input DNA, PCR amplification, examination in an agarose gel electrophoresis, manual normalization, and pooling.

We sequenced the samples in Illumina NextSeq 550 System with a High-Output Kit to generate PE reads of 150 bp. Since this system allows pooling libraries (Trevisan et al., 2019), we performed four sequencing experiments with different numbers of specimens (eight, 28, 33, and 33, respectively). We performed DNA sequencing in the Core Facility for Scientific Research-University of São Paulo (CEFAP-USP).

### Quality Control, Mitogenome Assembly, and Annotation

We used the HTQC toolkit (Yang et al., 2013) and an original *Python* script (selectTiles.py, see Machado et al., 2016) that automates tile selection to trim and filter the sequences. We used FASTQC (Andrew, 2010) to evaluate the quality of filtered reads. The assembly protocol received only filtered PE reads. Machado et al. (2016, Appendix S1) describes the complete procedure.

To assemble the mitogenomes, we performed the baiting and iterative mapping strategy implemented in MIRA v4.0 (Chevreux et al., 1999, available at: https://www.chevreux.org/projects_mira.html) and a modified version of MITOBIM.PL v1.6 (Hahn et al., 2013, available at: https://github.com/chrishah/MITObim) following Machado et al. (2016, Appendix S2). The reference mitogenome sequence of the tapeworm *Rhinebothrium reydai* Trevisan and Marques, 2017 (GenBank Accession Number NC_044703.1) was the bait for the assembly. We verified whether the assembly generated a circular genome by using AWA (Machado et al., 2018, the AWA beta version is available at https://gitlab.com/MachadoDJ/awa). We used Bowtie2 v2.2.6 (Langmead and Salzberg, 2012, available at: http://bowtie-bio.sourceforge.net/bowtie2/index.shtml) to map the raw reads back to the putative mitogenome selected by AWA using the local alignment algorithm and the highest sensitivity setting, with the threshold for base calling on the consensus sequence to bases that match at least 99%. Finally, in order to refine ambiguous regions, we submitted the sequences to Pilon v1.23 (Walker et al., 2014, available at: https://github.com/broadinstitute/pilon), which is recommended to polish *de novo* assemblies from short read data (e.g., Illumina).

**FIGURE 1:**
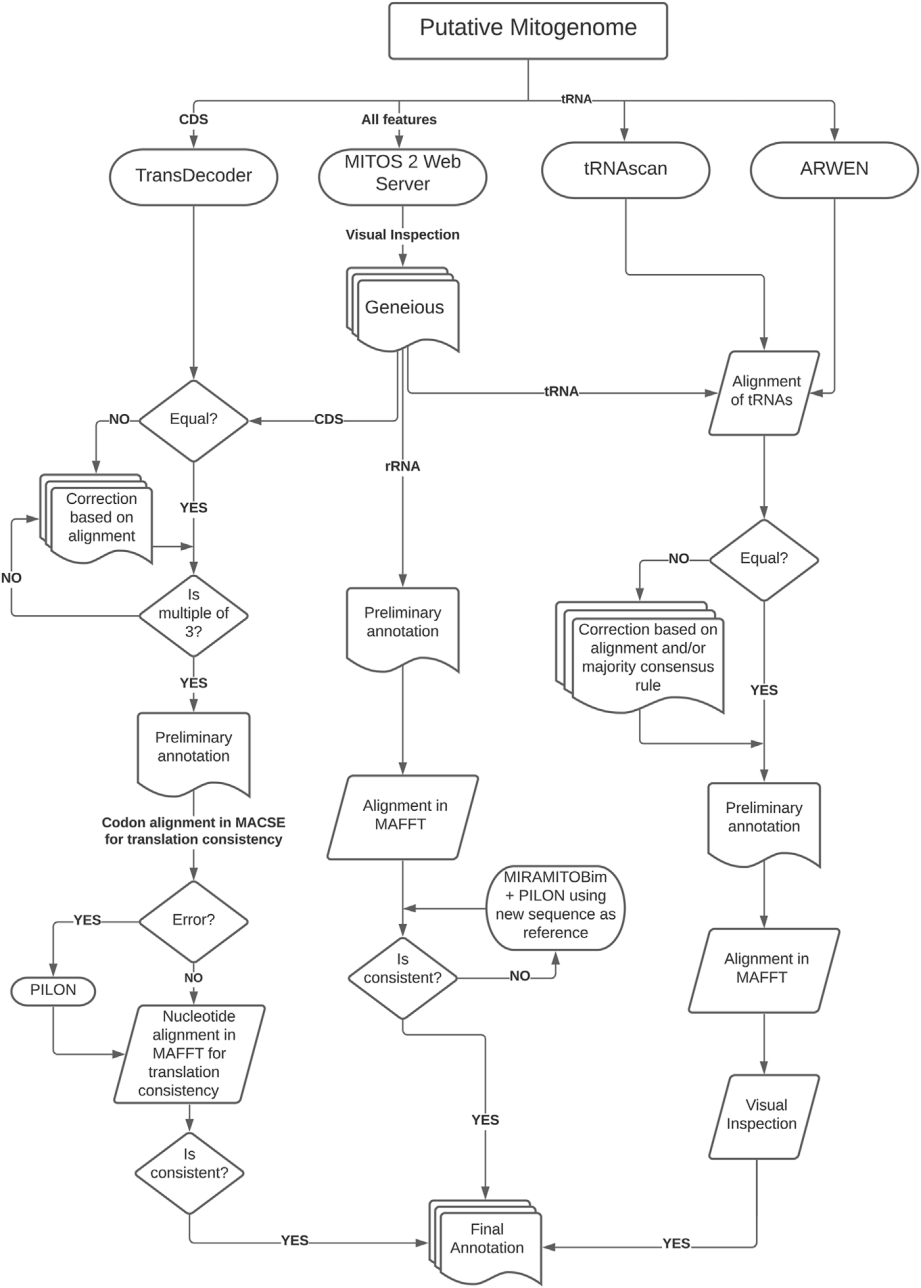
Schematic workflow of the annotation protocol used in this study.

Assembled mitogenomes were initially annotated using MITOS2 webserver (genetic code table = 9) (Bernt et al., 2013, available at http://mitos2.bioinf.uni-leipzig.de) for preliminary annotation. After that, we followed a more complex strategy to verify the results since MITOS2 has limited reference sequences for cestodes. This limitation results in some misplaced start or end positions or even the lack of annotation of some genes, which requires a time-consuming manual curation (Trevisan et al., 2019). Therefore, we used three different strategies to curate the annotation of coding genes, rRNAs, and tRNAs, for which the workflow of the annotation protocol is depicted in Figure 1.

To predict coding genes, we used TransDecoder (see Hahn et al., 2013, available at https://github.com/TransDecoder) to identify candidate coding regions which were compared with the output from MITOS2 after visual inspection in Geneious. After that, we checked for inconsistencies (e.g., presence of INDELs and stop codons in the middle of the sequences) and if sequence length were multiples of three. Then we translated each CDS on MACSE (Ranwez et al., 2011; using the echinoderm and flatworm mitochondrial code, available at: https://mbb.univ-montp2.fr/MBB/subsection/softExec.php?soft=macse2) to check for translation errors and stop codons. After that, we used MAFFT—Global Alignment v7 (Katoh et al., 2002, available at: https://mafft.cbrc.jp/alignment/software/) to align putative homologous regions and AliView v1.26 (Larsson, 2014, available at: https://ormbunkar.se/aliview/) to visualize the alignment. This step was essential to detect and correct unusual INDELs by running the sequence through Pilon or, in some cases, reassembling the regions using the preliminary assembly as bait in MIRA/MitoBIM and Pilon performing a visual inspection in AliView.

To annotate the transfer RNA (tRNA) sequences, we used ARWEN (Laslett and Canbäck, 2007, available at: http://130.235.244.92/ARWEN/) and tRNAscan-SE (Lowe and Eddy, 1997; Schattner et al., 2005, available at: http://lowelab.ucsc.edu/tRNAscan-SE/). The outputs from these programs were compared visually with MITOS2 output to extract the putative tRNAs. If they were not equal, we corrected them based on alignment or the majority consensus rule. Then we realigned the regions in MAFFT and visually inspected them to check for inconsistencies (e.g., presence of INDELs). The ribosomal RNA (rRNA) sequences were annotated based on MITOS2 output aligned on MAFFT to check for inconsistencies. If necessary, we reassembled problematic regions using the initial region as bait (running MIRA/MitoBIM and Pilon) and performed visual inspections in AliView. We annotated the non-coding region (NCR) with sequence similarity searches in BLAST using default parameters. The secondary structures of the tRNAs were predicted with MITOS2 and RNAfold (Hofacker, 2003, available at: http://rna.tbi.univie.ac.at/) and posteriorly edited on Inkscape v1.0.2 (available at: http://www.inkscape.org/) to highlight the variable loop region.

### Informative Character Analysis

We estimated the information content on each tRNA, rRNA, and protein-coding gene in the set of 88 tapeworm mitogenomes (86 from the present study and two sequences from GenBank -NC_044664 and NC_044703). Character information content depends on the optimality criterion used. We considered informative characters under the parsimony criterion as any character with two or more character states for which each character state has to be represented by at least two terminals.

For instance, given five terminals, characters with the following states AAAAA, AAAAC, or AATGC are not informative while a character with AACCT is. Next, we pooled nucleotide sequences of tRNA and rRNA genes. For protein-coding genes, we pooled their respective amino acid sequences. This resulted in 36 multi-FASTA files (12 protein-coding genes, two rRNA genes, and 22 tRNA genes). Sequences in each multi-FASTA file were aligned using the argument “--auto” in MAFFT v7.475.

We transplanted gaps from amino acid alignments into their respective nucleotide sequences to generate nucleotide alignments that correspond to the amino acid alignments and represent translation-based nucleotide alignments. We counted all the informative characters from the resulting 36 nucleotide alignments (following the criteria described above).

We calculated the frequency of informative characters per gene by dividing the number of informative characters by the alignment length. We calculated the correlation between the frequency of informative characters and the alignment length in R (R Core Team, 2014; v4.0.4) using the “lm” function, which serves to fit linear models. Finally, we plotted the correlation data using “stat_smooth.”

We calculated the rate of non-synonymous to synonymous mutations (d*N*/d*S*) using the sequence *Anthocephalum* sp. BU001 (GenBank MZ594567) as reference. The d*N*/d*S* ratio quantifies the mode and strength of selection by comparing synonymous substitution rates (d*S*)—assumed to be neutral—with non-synonymous substitution rates (d*N*), which are exposed to selection as they change the amino acid composition of a protein (see Mugal et al., 2014 for a discussion on codon evolution and the temporal dynamics of d*N*/d*S*). We also calculated the correlation between the frequency of informative characters and the d*N*/d*S* ratio.

Finally, we computed the observed codon usage (i.e., the number of different codons used in protein-coding genes) and its variation according to observed GC3% (i.e., the GC content of the third positions of each codon). We disregarded the start and stop codons of each sequence in our analysis to remove the bias caused by their insertion.

## Results

### Mitogenome Organization and Structure

**FIGURE 2:**
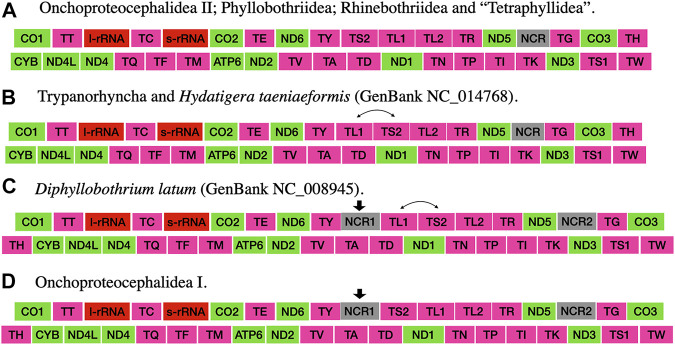
Gene order of the mitogenomes presented in this study and those cited in the discussion section (taken from Li et al., 2017). Arrows indicate the acquisition of a feature. Double-headed arrows indicate the swap of two features.

**FIGURE 3:**
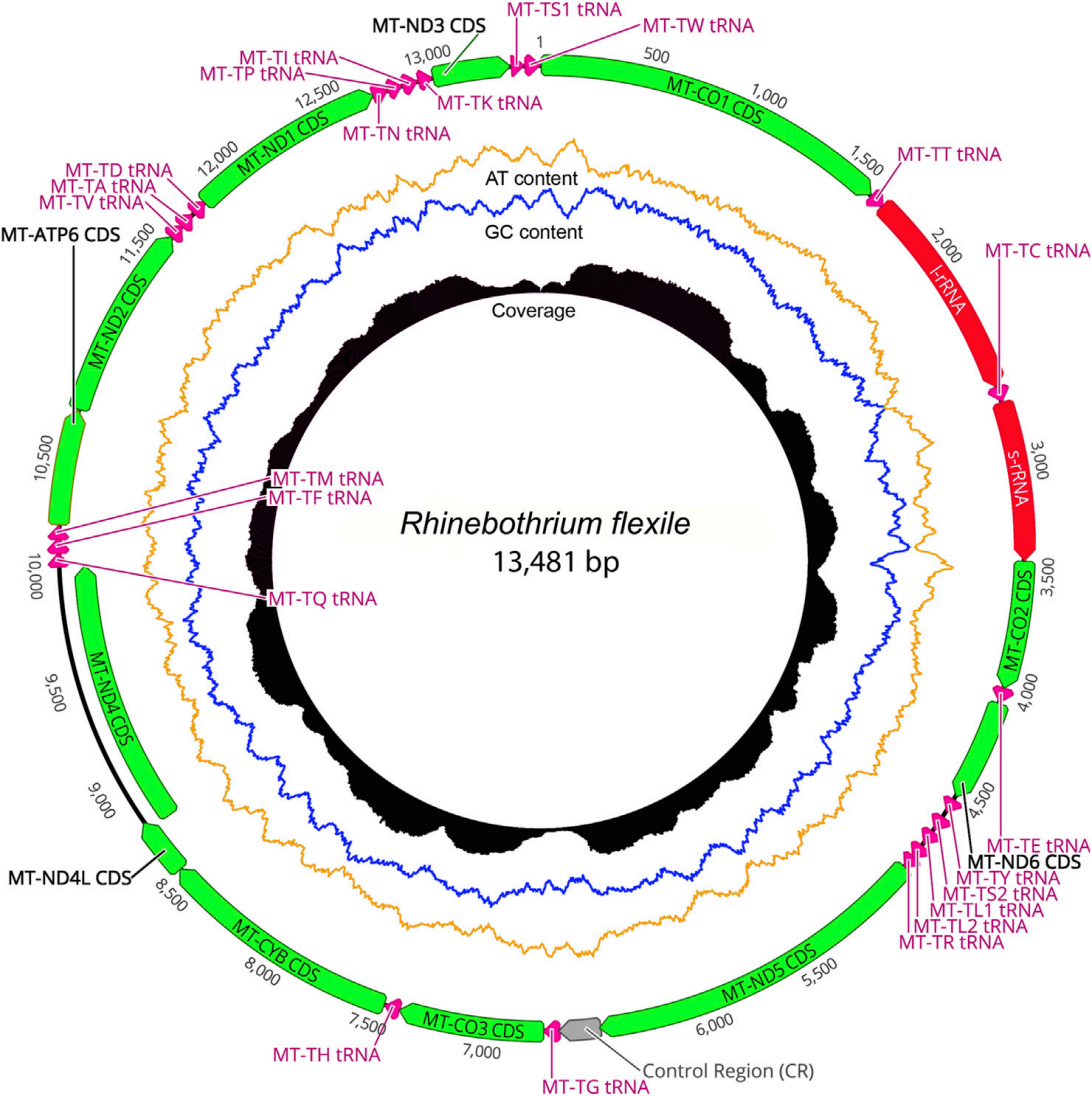
The complete mitogenome map of the *Rhinebothrium flexile*—BU006 (GenBank MZ594571), selected to represent the gene organization of the new mitochondrial genomes described. The image was generated with Circleator v1.0.2 (Available at: http://jonathancrabtree.github.io/Circleator/). Grey: control region; green: genes; red: rRNA; pink: tRNA; AT%: yellow; GC%: blue. Coverage (black skyline plot) shows coverage values generated by mapping the reads on the mitogenome sequence with Bowtie2 (Langmead and Salzberg, 2012).

The taxonomic representation of this study is comprised of members from five orders of cestodes: Onchoproteocephalidea II (sensu Caira et al., 2014b) (five specimens); Phyllobothriidea (six specimens); Rhinebothriidea (72 specimens); “Tetraphyllidea” (two specimens), and Trypanorhyncha (one specimen). We listed the details of each specimen sequenced in Supplementary Table S3, together with data of their hosts, collection localities, GenBank, and voucher accession numbers. The complete mitogenomes from the 86 specimens are circular, encoded in the same strand, and transcribed in the same direction. Mitogenome sizes varied from 13,369 to 13,795 bp, followed the pattern observed previously for tapeworms (13–15 kb; see Li et al., 2017; Trevisan et al., 2019). The mean sequence depth of each mitogenome varied from 66.67 to 2,352.51 bp. There are 36 genes in each mitogenome, including 12 protein-coding genes (MT-ATP6, MT-CO1–3, MT-CYB, MT-ND1–6, and MT-ND4L), 22 tRNAs, two ribosomal RNA genes (RNR1-2), and one control region (NCR). As expected, the ATP8 gene was not found, which is consistent with findings for Neodermata, despite its presence in other metazoan mitogenomes (Le et al., 2002; Guo, 2016; Zhao et al., 2016; Egger et al., 2017; Li et al., 2017). Except for a few tRNA rearrangements and the number of control regions (NCR; i.e., one), the gene order in all specimens follows the typical organization of cestodes (Li et al., 2017; Trevisan et al., 2019) (Figure 2). Therefore, we provide the complete mitogenome map of *Rhinebothrium flexile* (GenBank MZ594571) (Figure 3), to represent the gene organization of the new mitochondrial genomes described. The complete annotation; general statistics including length, skewness, and A/T content (%) of the protein-coding genes (CDs), tRNAs and rRNA genes; each codon position of CDs and non-coding region (NCRs), and GenBank accession numbers for each of the 86 mitogenomes is available in Supplementary Table S4. This supplementary table also includes coverage information for each mitogenome.

### Protein Coding Genes and Codon Usage

Sizes of 12 coding genes varied from 261bp (ND4L) to 1,629 bp for the Onchoproteocephalidea II, 1,602 bp for the Phyllobothriidea, 1,647 bp for the Rhinebothriidea, 1,608 bp for the “Tetraphyllidea” for MT-CO1, and 1,572 bp for the Trypanorhyncha for both MT-CO1 and MT-ND5 (Supplementary Table S5). Genes commonly showed size variation among species (Supplementary Table S3), with the exception of four of them: MT-ATP6 (516 bp), MT-CO2 (570 bp), MT-CO3 (675 bp), and MT-ND4L (261 bp) (Supplementary Table S5). Start and stop codons (ATG/GTG and TAG/TAA, respectively) were found to be those most commonly reported for cestodes (Li et al., 2017; Trevisan et al., 2017; Landeryou et al., 2020) (Supplementary Table S5). The start codon ATG seems to be the most frequent (Onchoproteocephalidea II—91.6%; Phyllobothriidea—90.3%, Rhinebothriidea—92.4%; “Tetraphyllidea”—91.6% and Trypanorhyncha—100%) (Supplementary Table S5). The gene MT-CO3 has GTG as start codon in all taxa with the exception of Trypanorhyncha and for 25 out 72 specimens of Rhinebothriidea (Supplementary Table S3). Rhinebothriideans also have GTG as start codon for MT-ND5 (8 specimens), MT-ND4L (4 specimens), MT-ND3 (3 specimens), MT-ND4 (2 specimens) and, MT-ND1 and MT-CO1 with one specimen each (Supplementary Table S3). The differences for the Rhinebothriidea could be related to its larger taxonomic representation in comparison to other taxa, which allowed us to detect codon variation. For stop codons, TAA is most frequently used for the Onchoproteocephalidea II (73.3%), Rhinebothriidea (53.9%), and Trypanorhyncha (58.3%); and TAG is the most used for the Phyllobothriidea (58.2%) and Tetraphylidea (62.5%) (Supplementary Table S5). The A/T content from the orders is within the range reported previously for cestodes (i.e., 65.6–76.5% vs. 58.6–76.6 from Li et al., 2017; Li et al., 2018; Trevisan et al., 2019; Landeryou et al., 2020) (Supplementary Table S5). Overall, the three most used family codons for the orders are T-rich, as Leucine, Phenylalanine, and Serine (in this order), which are commonly reported for cestodes (Li et al., 2017; Li et al., 2018; Trevisan et al., 2019; Landeryou et al., 2020). The only exception is the Trypanorhyncha that also possesses Valine as the third most used, tied with Phenylalanine (Supplementary Table S5).

### Transfer and Ribosomal RNAs

The 22 tRNA genes expected for the mitogenome of cestodes were identified, ranging from 47–76 bp (47–73 for Rhinebothriidea, 47–76 for “Tetraphyllidea,” 51–70 for Onchoproteocephalidea II, 54–70 for Phyllobothriidea, and 58–68 for Trypanorhyncha; Supplementary Table S2).

**FIGURE 4:**
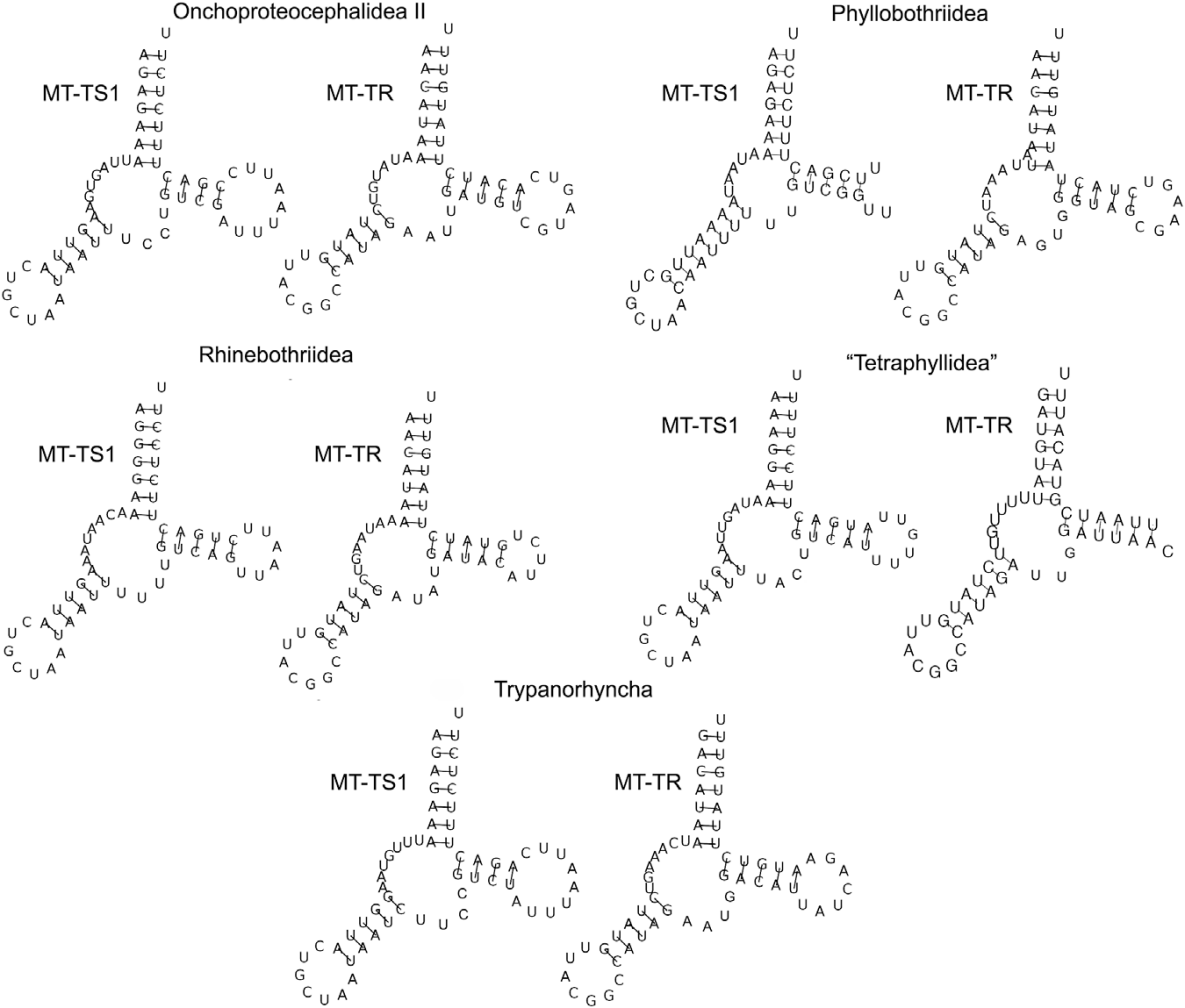
The secondary structure of MT-TS1 and MT-TR from five orders of cestodes (one specimen representing each order) illustrates the lack of DHU-arms found for these tRNAs.

The secondary structure of each tRNA is folded into the traditional cloverleaf structure, with the exception of MT-TS1 and MT-TR, which lacked DHU-arms (Figure 4).

All tRNAs had the standard anticodons, except for the MT-TR. Even though two representatives of the Echeneibothriidae have the common TCG as MT-TR anticodon, the other 84 specimens possess an anticodon of ACG, which was also reported for the Diphyllobothriidea (Park et al., 2007; Li et al., 2017; Li et al., 2018). The order Caryophyllidea possesses a different anticodon for MT-TS1 in comparison to other cestode orders (TCT), except by *Khawia sinensis,* which have the same anticodon that was reported here (GCT) (Li et al., 2017; Xi et al., 2018).

The large ribosomal RNA gene (RNR1/16S) is located between MT-TY and MT-TC, with the small ribosomal gene (RNR2/12S) located between MT-TC and CO2 in all specimens (Figure 2). This gene order is conserved across all cestode orders (Li et al., 2017; Li et al., 2018; Trevisan et al., 2019; Landeryou et al., 2020). The length of the RNR1 varies from 952 to 1,061 bp (952–1,061 bp for Rhinebothriidea, 954–955 bp for “Tetraphyllidea,” 955–960 bp for Onchoproteocephalidea II, 959–970 bp for Phyllobothriidea, and 964 bp Trypanorhyncha). The range for the RNR2 is smaller in the number of base pairs 677–737 bp (677–737 bp for Rhinebothriidea, 708–712 bp for Onchoproteocephallidea II, 709–710 bp for “Tetraphyllidea,” 712–719 bp for Phyllobothriidea, and 720 bp for Trypanorhyncha) (Supplementary Table S5). The mean of the concatenated size of the RNR1-2 for each order is 1,664 bp for Tetraphyllidea, 1,667 bp for Onchoproteocephalidea II, 1,674 bp for Rhinebothriidea, 1,684 bp for Trypanorhyncha, and 1,703 bp for Phyllobothriidea. The A/T content in rRNAs varies from 65.5 to 71.6% across the five orders (Supplementary Table S5).

### Non-coding Regions

All assembled mitogenomes presented a single NCR flanked by MT-ND5 and MT-TG (Figures 2A,B, Supplementary Table S4). To date, most cestode mitogenomes available possess two NCRs (Li et al., 2017; Li et al., 2018; Lenderyou et al., 2020). However, *Pseudanoplocephala crawfordi*, *Taenia crocutae*, *Taenia solium,* and *S. acheilognathi* have been reported to possess three NCRs (Li et al., 2017), and *Hydatigera taeniaeformis*, *Rhinebothrium reydai* and *Anindobothrium anacolum* (Trevisan et al., 2019) to have only one NCR. Despite the variation in the number of NCRs, their location is standard for six orders: Cyclophyllidea, Diphyllobothriidea, Onchoproteocephalidea II, Phyllobothriidea, Rhinebothriidea, and Trypanorhyncha. However, the position NCRs in Caryophyllidea and Botriocephallidea differs from the others (Li et al., 2017; Li et al., 2018; Trevisan et al., 2019; Landeryou et al., 2020).

The NCR’s length varies from 177 to 294 bp (177–294 bp for Onchoproteocephalidea II, 125–244 bp for Phyllobothriidea, 86–318 bp for Rhinebothriidea, 187–288 bp for Tetraphyllidea, and is 127 bp for Trypanorhyncha) which follows the previous lengths reported for other cestodes (Li et al., 2017; Li et al., 2018; Trevisan et al., 2019; Landeryou et al., 2020) (Supplementary Table S5). All orders reported in this study showed a high A/T content bias for the NCR region in comparison to the average of their entire sequence: 81.3 vs. 71.2% for Onchoproteocephalidea II; 79.3 vs. 70.3% for Phyllobothriidea; 80.1 vs. 67.5% for Rhinebothriidea; 75.6 vs. 66.1% for Tetraphyllidea; and, 82.7 vs. 70.5% for Trypanorhyncha (Supplementary Table S5), corroborating the findings of Li et al. (2017; 2018) and Landeryou et al. (2020).

### Informative Characters

**FIGURE 5:**
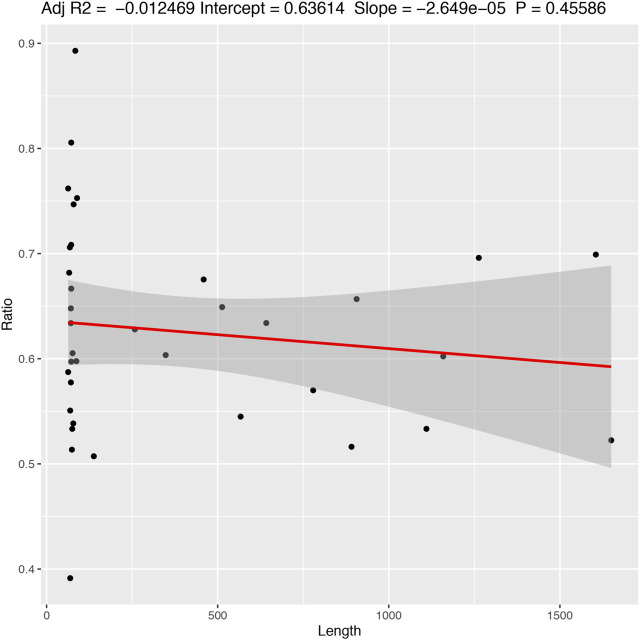
Correlation between the frequency of informative characters and sequence length. Adjusted R2 = −0.012469; Intercept = 0.63614; Slope = −2.649e−05; *p*-value = 0.45586.

**FIGURE 6:**
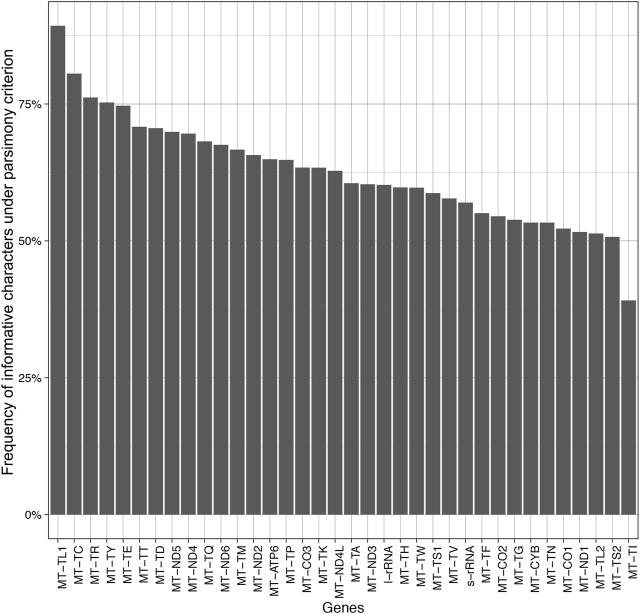
Barplot of the frequency of informative characters under the parsimony criterion.

The results indicate that there is little or (more likely) no correlation (adjusted R2 = −0.012 with a *p*-value = 0.455) between the length of the alignment and the number of informative characters (Figure 5). Additionally, the results suggest a variation in the number of informative characters across groups of genes (Figure 6).

**FIGURE 7:**
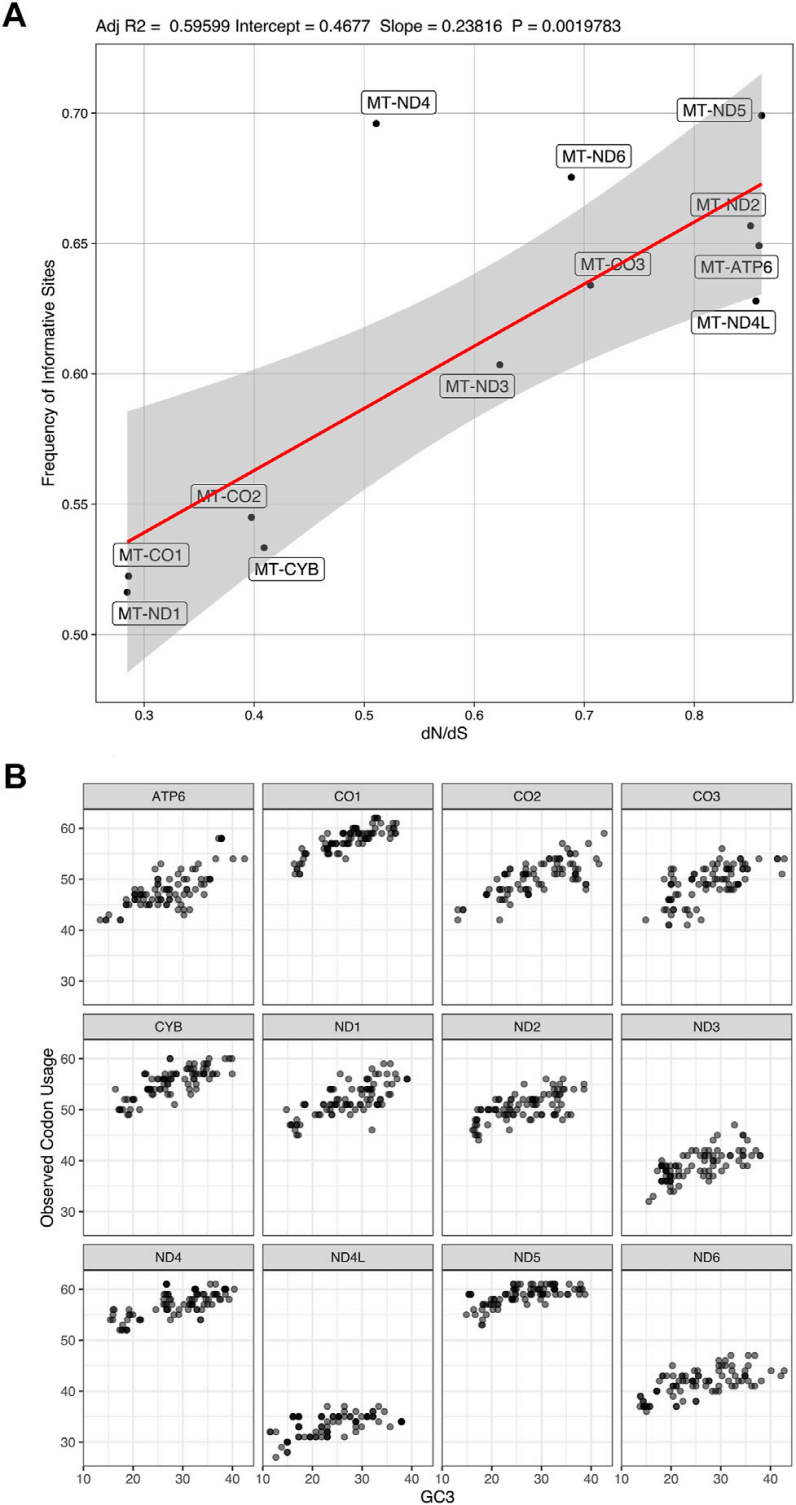
Frequency of informative sites and codon usage. **(A)**. correlation between the frequency of dN/dS mutations and number of informative sites (Adjusted R2 = 0.59599; Intercept = 0.4677; Slope = 0.23816; *p*-value = 0.0019783). **(B)**. codon usage variation among the mitochondrion coding genes in relation to content in the third codon position.

There is a correlation (adjusted R2 = 0.595 with a *p*-value = 0.002) between the rate of non-synonymous/synonymous mutations (d*N*/d*S*) and the number of informative sites from coding genes (Figure 7A). We found a variation in codon usage to GC3% among the mitochondrion coding genes (Figure 7B), in which MT-ND5 uses more codons in comparison to other CDs.

The mitochondrially encoded MT-TL1 (63–68 bp in length) contains the highest frequency of informative characters in our dataset (89.29%) (Figure 6). It is followed by MT-TC and the MT-TR that possess 80.56 and 76.19% of informative characters, respectively. However, tRNA genes do not necessarily contain more informative sites than all other genes in this dataset. The three genes with the lowest frequency of informative characters are also tRNA genes (Figure 6). The genes MT-TL2, MT-TS2, and MT-TI contain 51.35, 50.72, and 39.13% informative characters, respectively. Among the protein-coding genes, MT-ND5 and MT-ND4 have the highest frequency of informative characters (69.91 and 69.60%, respectively), while MT-CO1 and MT-ND1 have the lowest frequency of informative characters (52.24 and 51.63%, respectively) (Figure 6).

## Discussion

### Mitogenomes Structure and Comparison Within Cestode Orders

The complete mitogenomes provided here represent an increase of 41% of the mitogenomes available for cestodes (61–147) and an addition of 16% in the taxonomic representation of the cestode orders (six to nine of 19 orders). This study contains the first report for the Phyllobothriidea, Onchoproteocephalidea II, “Tetraphyllidea,” and Trypanorhyncha. This is also the first report of mitogenomes for 14 nominal species and new species that need to be formally described. Within the Rhinebothriidea, there were only two specimens for which the mitogenome had been sequenced to date (Trevisan et al., 2019), representing only two of five families recognized for the order: Rhinebothriidae (i.e., *Rhinebothrium reydai*) and Anindobothriidae (i.e., *Anindobothrium anacolum*)—GenBank Acc Number NC_044703 and NC_044664, respectively. We included specimens for all five families of Rhinebothriidea (55 Rhinebothriidae; nine Anthocephaliidae; three Anindobothriidae; and two Echeneibothriidae and Escherbothriidae, respectively). For the family Rhinebothriidae, we obtained the mitogenome for six of the 10 genera currently recognized (Ruhnke et al., 2015; Coleman et al., 2019), including two mitogenomes of the type species: *Rhinebothrium flexile*.

The nucleotide composition of the mitogenomes presented here reinforces the bias towards A and T documented for cestodes (Li et al., 2017; Trevisan et al., 2019; Landeryou et al., 2020), with A/T content varying from 63.4 to 73.4%. Furthermore, Our results reinforce that the gene order in the cestode mitochondrion is highly conserved (Nakao et al., 2003; Li et al., 2017; Zhang et al., 2017; Trevisan et al., 2019).

The concatenation of the tRNAs of the mitogenomes allowed us to estimate the average length of tRNAs varied from 1,407 to 1,424 bp for each mitogenome (Supplementary Table S4), which follows the pattern observed previously (Li et al., 2017; Landeryou et al., 2020). The absence of DHU-arms for those two tRNAs has also been reported for Bothricephallidea, Caryophyllidea, Cyclophyllidea, Onchoproteocephalidea I, and Diphyllobothriidea (Park et al., 2007; Li et al., 2017; Xi et al., 2018; Li W. X. et al., 2019). The deletion of the DHU-arm in MT-TS1 was expected since it is commonly reported for other metazoan mitogenomes (Garey and Wolstenholme 1989; Lavrov et al., 2000; Jühling et al., 2012; Yoon and Park, 2015). This event has been suggested as an indication that it occurred early in the diversification of metazoans (Garey and Wolstenholme 1989; Lavrov et al., 2000; Jühling et al., 2012; Pons et al., 2014; Yoon and Park, 2015).

Our results corroborate the findings of Trevisan et al. (2019) that the protocols adopted in this study are suitable for obtaining molecular data with different levels of preservation and a low amount of input DNA. We recovered the complete mitogenome of 86 specimens, regardless of their fixation age, which varied within the years 1996–2019. The mean coverage obtained ranged from 66.67 to 2,352.51 bp, which allowed us to detect the 36 features expected for their mitogenomes. Their sizes (about 13 kb) followed the pattern previously observed for tapeworms (13–15 kb) (Li et al., 2017; Trevisan et al., 2019).

The gene order of cestodes is highly conserved (Nakao et al., 2003; Li et al., 2017; Zhang et al., 2017; Trevisan et al., 2019), but there are rearrangements of some tRNA genes and variation in the number of control regions (one to three) among the orders (Li et al., 2017; Trevisan et al., 2019). Li et al. (2017) speculated that there should be more gene rearrangements in other orders of cestodes, which needed further investigation with the description of new mitogenomes. Trevisan et al. (2019) also identified the need to compile other mitogenomes of cestodes to better understand the rearrangement events and the number of NCRs associated with the group’s diversity. Our results corroborate the assumption that sharing MT-TS2−MT-L1 is a putative synapomorphy for the acetabulate cestodes (Trevisan et al., 2019) since all specimens but Trypanorhyncha possess the same gene order (i.e., Category IV from Li et al., 2017). However, we should point out that this condition has been reverted to the ancestral state (MT-TL1–MT-TS2) in taeniids.

All mitogenomes assembled in the present study presented only one NCR. This condition was only known for the cyclophyllidean *Hydatigera taeniformis* (Li et al., 2017) and two species of rhinebothriideans (Trevisan et al., 2019). If we consider the position of the NCR to other genes, all mitogenomes with only one NCR possess the same gene order except for the trypanorhynch and the cyclophyllidean *Hydatigera taeniformis*, which possess the same gene rearrangement (Figure 2). This could indicate that the NCR position relative to other genes could also be a putative synapomorphy for acetabulated cestodes (reverted in *Hydatigera taeniformis*).

The gene order found in specimens of Onchoproteocephalidea II is different from the published for two species of Onchoproteocephalidea I (*Testudotaenia* sp. GenBank KU761587; and *Gangesia oligonchis* GenBank MF314173) (Li et al., 2017; Li W. X. et al., 2019). The difference is in the presence of one additional NCR between tRNA-Tyr and tRNA-Ser2 reported by the authors (Figures 2A,D, respectively). This additional NCR is questionable since this is not a reference sequence (RefSeq) from GenBank and considering that there are specific challenges involved in annotating the mitogenomes of cestodes (Trevisan et al., 2019), including manual curation. Hence, we suggest that more specimens of Onchoproteocephalidea I and II be sequenced to confirm their gene order and number of NCR.

### Is There an Ideal Mitochondrial Marker for Cestodes?

Accessing the information content of genes across mitogenomes could reveal target regions for phylogenetic studies and unveil important information related to biological processes, such as the mutation rates. Therefore, we measured the informational content, d*N*/d*S* ratio, and codon usage of all sampled mitochondrial genes (Figures 5–7).

We observed a greater frequency of informative sites on tRNAs genes (Figure 6). However, tRNAs tend to have small sizes compared to other genes (47–76 bp vs. >261 bp, respectively) (Figure 5). Also, we saw a high variation of informative sites among taxa for all genes (Figure 6).

We noted that MT-CO1 (1,569–1,647 bp in length) exhibited similar information content as MT-ND1 (891 bp in length) (52,24% vs. 51,63%, respectively) and that all other coding genes and rRNAs have greater frequencies of informative sites (Figure 6) in comparison to MT-CO1. Thus, the informational content of other mitochondrial genes in the cestode genome is similar or higher than the informational content of MT-CO1.

We also observed variations of the d*N*/d*S* ratio among mitochondrial coding genes. There could be a weak positive correlation between the d*N*/d*S* ratio and the information content of coding genes (adjusted R2 = 0.596 with *p*-value = 0.002; see Figure 7A). Also, codon usage variation to GC3% is not the same among all mitochondrion coding genes (Figure 7B). Despite the possible correlation between informational content and d*N*/d*S*, it is clear that all 12 mitochondrial coding genes of the sampled tapeworms are under different selective pressures and accumulate phylogenetic information at different rates. We infer that no single coding gene can provide the same level of phylogenetic information as all coding genes were taken together.

Despite the observed variation among mitochondrion genes, MT-CO1, MT-ND1, and MT-CYB are the most frequently sequenced for cestodes (Supplementary Table S6), and MT-CO1 seems to be the marker of choice in many phylogenetic studies in the group. Some authors have proposed the choice of a few molecular markers for cestodes. For example, Littlewood et al. (2008) proposed that rRNR1 and MT-ND1 are more informative for Cyclophyllidea. Zarowiecki et al. (2007) suggested MT-ND4, MT-COX3, and MT-ND4L as preferred molecular markers for the genus *Schistosoma* (Digenea).

Some authors could suggest that genes such as MT-ND5 (informative sites = 69.91%, dN/dS = 0.861) would be a better potential molecular marker for the observed taxa because of their dN/dS ratios that approach 1.0 and their high informational content (Ford, 2002; Holderegger et al., 2006; Marshall et al., 2009). However, there is no consensus on the literature about the dN/dS ratio for phylogenetic markers, and we lack evidence that specific genes would be inadequate for phylogenetic analysis due to their dN/dS ratio.

For example, Roje (2014) discusses some assumptions frequently present in studies that genes such as Rhodopsin could be unsuited for phylogenetic analysis of certain aquatic organisms (mainly fish) because it evolves under strong positive selection (dN/dS >> 1.0). However, their results showed that neutrality alone (dN/dS ≈ 1.0) does not determine congruence in topology, and those data that are inferred to have evolved under selection should not necessarily be excluded. Others, as Mekvipad and Satjarak (2019), for instance, state that low dN/dS ratios indicate relatively high conservation levels. Hence, they argue that genes with low dN/dS ratios could be “particularly good” candidates for phylogenetic markers of chlorophyte algae. However, our results seem to contradict this assumption. As illustrated in Figure 7A, lower dN/dS ratios are associated with lower phylogenetic information content.

Given that the specialized literature does not have a consensus opinion about the ideal dN/dS ratio for phylogenetic reconstruction, it appears unwise to discard markers based on the inferred evolutionary pressures based on this single parameter. Furthermore, the ample variation of informational content, dN/dS ratio, and codon usage among the observed sequences suggest that no single gene could tell the complete evolutionary history of the mitogenome and therefore indicate that no phylogeny based on any of these genes alone can encompass the entire evolutionary information stored in these mitochondria.

Total evidence analysis allows examining assumptions and creating a conjoint hypothesis of evolutionary relationships from different data sources, which increases the explanatory power of the analysis. One of the benefits of total evidence is the potential for complementary information from different markers or data sources that could support different areas of the cladogram (Nixon and Carpenter, 1996; Wiens, 1998; Pickett et al., 2005). Under this context, our results allow speculating that the poor resolution observed in cestodes phylogenies could be improved with phylogenetic analyses conducted under a total evidence framework. And, considering that it has become easier to obtain the complete information content of a locus (e.g., mitochondria) for a broader taxonomic representation. Thus, our results justify the sequencing, assembly, and annotation of the entirety of the cestode mitogenome, suggesting that for these datasets, no molecular markers should be used as a “silver bullet.”

## Conclusion

Considering the number of terminals sequenced, this is the most comprehensive documentation of mitogenomes of cestodes to date. This is also the first report of mitogenomes for the orders Phyllobothriidea, Onchoproteocephalidea II, “Tetraphyllidea” and Trypanorhyncha, and also, the first report of mitogenomes for 14 nominal species and for new species that need to be formally described. The taxonomic representation achieved in this study was only possible due to de development of methods and analytical strategies outlined by Trevisan et al. (2019). Our results suggest that no single gene should be used as a molecular marker alone since none could tell all the evolutionary history in the mitogenome and that cestode phylogenies should be improved with phylogenetic analyses conducted under a total evidence framework. Therefore, the characterization of the 86 new mitochondrial genomes is the first step to provide a useful source for future studies on the evolutionary relationships of these groups. Thus, we encourage future studies to sequence more specimens from different cestode orders, considering the potential of the complete mitogenome and the rearrangement information (number and position of the NCR). The combination of these strategies followed by phylogenetic analyses can increase the power to test these hypotheses, providing a better understanding of the historical relationships within cestodes.

## Data Availability

The complete and annotated cestode mitogenomes produced here are available at NCBI’s GenBank, accession nos. MZ594567-MZ594652, and BankIt, accession no. 2483023. The data sequenced for this study have been deposited in NCBI’s Sequence Read Archive (SRA) through the BioProject accession no. PRJNA484227. The corresponding BioSample accession nos. are SAMN17771650-SAMN17771735.

## Supplementary Material

Supplementary Table S1 List of the 61 cestode mitochondrial genomes used for comparative analyses in this study.

Supplementary Table S2 Details on the hosts examined in the present study. GCD is the Global Cestode Database. For additional details on the host, access https://tapewormdb.uconn.edu/ and search for the correspondent host field number in elasmobranch specimens.

Supplementary Table S3 Taxonomic representation of the specimens analyzed in the present study, their ordinal and family placement; specimen and voucher number; Genbank number; host identification; and locality.

Supplementary Table S4 Annotation, general statistics, and GenBank accession numbers for each of the 86 mitogenomes presented in this study. General statistics include length, skewness, and A/T content (%) of the protein-coding genes (CDs), tRNAs, rRNA genes, each codon position of CDs, and non-coding region (NCRs). This table also includes coverage information for each mitogenome.

Supplementary Table S5 General statistics for the mitogenomes of the five orders presented in this study. General statistics include length, skewness, and A/T content (%) of the protein-coding genes (CDs), tRNAs, rRNA genes, each codon position of CDs, and non-coding region (NCRs).

Supplementary Table S6 The approximate number of mitochondrial genes available at NCBI. Data compiled on June 3rd, 2021.
